# Scientific Collaborations: How Do We Measure the Return on Relationships?

**DOI:** 10.3389/fpubh.2016.00009

**Published:** 2016-02-15

**Authors:** Jeanne M. Fair, Martha Mangum Stokes, Deana Pennington, Ian H. Mendenhall

**Affiliations:** ^1^Cooperative Biological Engagement Program, Defense Threat Reduction Agency, Fort Belvoir, VA, USA; ^2^Department of Geological Sciences, University of Texas at El Paso, El Paso, TX, USA; ^3^Duke-NUS Graduate Medical School, Singapore, Singapore

**Keywords:** scientific collaboration, return on relationships, Hantavirus, MERS, systems dynamics

## Abstract

Emerging infectious diseases (EIDs), the majority of which are zoonotic, represent a tremendous challenge for public health and biosurveillance infrastructure across the globe. Due to the complexity of zoonotic pathogens, it is essential that research and response to EIDs be a transdisciplinary effort. And while crisis and circumstance may be the initial catalyst for responding to an outbreak, we provide examples of how transdisciplinary scientific collectives, which are organized and solidified in advance of crises, can transform the way the world responds to outbreaks and in some cases could even prevent one from occurring (1). Current methods for assessing whether a cooperative engagement between countries is producing measurable and sustainable value is based on the ideas of return on investment and do not consider the inherent importance of relationships. In this article, we apply the idea of return on relationships (ROR) and propose a method for measuring ROR, using a system dynamics modeling framework commonly used in epidemiology. Tracking the numerous and diverse scientific collaborations that emerged from a training workshop for biosurveillance of bats held in Singapore in 2014, we apply a methodology for visualizing and measuring the relationship networks and outcomes that result. Additionally, the collaborative, multidisciplinary network that coalesced in response to the Hantavirus outbreak in New Mexico is 1993 is discussed as an example of the long-term benefits of ROR.

## Introduction

Emerging infectious diseases (EIDs) represent a tremendous challenge for public health and biosurveillance infrastructure across the globe and are primarily zoonotic (2). Because zoonotic diseases cross species as well as borders, their detection, diagnosis, and prevention require collaboration and communication between a greater diversity of sectors and require national, regional and international coordination (3). A One Health approach (http://www.onehealthinitiative.com) which views the emergence of zoonotic infectious disease as the dynamic interactions of wildlife, domestic animal, human, and environmental ecosystems is required if the various sectors can indeed work together in order to confront these pathogens. Understanding the complex interactions inherent in zoonotic disease requires the union of medical doctors, veterinarians, virologists, wildlife biologists, epidemiologists, and geographers, to name a few. But the very diversity and breadth of a multidisciplinary team can present challenges to communication and teamwork, particularly in the midst of crisis, when it may be too late to build trust and establish communication mechanisms (1). While there are decades of research on teamwork in a wide variety of settings, few studies consider the dynamics of building informal networks across disciplines and institutions to which result in existing infrastructures of relationships that can be called upon when pathogens emerge and questions need to be answered quickly and collaboratively. A cluster analysis of scientific collaborations in physics by Chompalov et al. (4) revealed that the variety of organizational formats of collaborative projects range from formal and bureaucratic to informal and participatory. Recent studies show that scientific collaboration across disciplines and institutions is growing (5, 6). However, most studies of scientific teams analyze either observed interactions between team members or products of teamwork, especially publications. A better understanding of how collaborations are initiated and sustained through time, and the resulting outcomes (other than publications) is needed. Understanding these processes would enable development of an infrastructure where active surveillance partners can more effectively communicate across EID themes, facilitating responses and interventions.

Both the time scale and the qualitative nature of long-term scientific collaborations pose a challenge to traditional methods of measuring their value and impact. Every event, from trainings, workshops, and conferences to the activities surrounding an outbreak response, requires an investment of both time and monetary resources. And the scientific process necessary to understand the complex systems of zoonotic diseases within the environment requires an investment of years, if not decades, to review what is known, develop ideas and hypotheses, collect and analyze data, and continuously build upon a body of knowledge as new data emerge But assessing the value provided by long-term collaborations, whether those partnerships lead to research outputs, training activities, or simply the sharing of information and best practices, needs to occur within a shorter time scale, if the recognized value to be realized is to inform programmatic and resource allocation decisions. In order to assess the value in that time frame, we need to have a measurement of both the collaborative effort as well as its impact on current disease threat reduction systems. Current indicators that a cooperative engagement or a single event or workshop will produce outputs that are valuable and sustainable are based the measurement of return on investment (ROI) using metrics, such as the number of people trained or number of presentations. In an effort to respond to shrinking scientific budgets, more emphasis is being placed on ROI metrics for applied scientific programs, such as cooperative engagement programs. But the existing ROI metrics, which do not take into consideration the importance of relationships, are not suited for measurement of such intangibles. And how do we demonstrate that investment into the creation and fostering of relationships which are long-lasting, creative, and committed to solving problems can produce truly transformational outcomes which reduce the threat of infectious disease?

The need to develop a framework that captures the impact and benefit from scientific engagements is clear. Policymakers and Program Managers alike need to be able to evaluate when research and training activities are an efficient and effective use of federal dollars, and whether these activities help meet program objectives. But there are challenges to defining the appropriate metrics, including aligning long project timelines with shorter programmatic milestones. Traditionally, the focus has been on reporting research results rather than measuring broader benefits and the synergy of those metrics across different types of activities.

Here, we propose a methodology for visualizing and quantifying the outcomes of such collaborations. We employed the idea of return on relationships (ROR) and propose a method for measuring ROR using a systems dynamics modeling framework. We modify the definition of ROR commonly used in business, as *the long-term net outcome emerging for all parties resulting from the establishment and mutual maintenance of a relational engagement* (7). It is implied that a ROR is an outcome of a mutually reciprocal process and can be assessed on a relationship level, in addition to each member of the relationship (8). Like business, cooperative engagements are reciprocal in nature and may also be appropriate to measure ROR.

**Figure 1:**
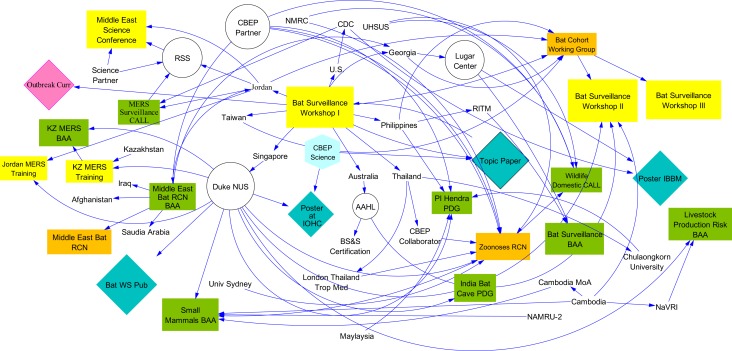
**System dynamics model of tracked outcomes of the training workshop (e.g., countries, institutions, and endpoint metrics, such as publications, presentations, and proposals)**.

Using the scientific collaborations that emerged from the training workshop for biosurveillance of pathogens in bats held in Singapore in 2014, we apply a methodology for visualizing and measuring the resulting relationship networks and outcomes (Figure 1). The results of our analysis show that system dynamics principles can be applied to networks of relationships and the resulting information can then be used to measure the impact of efforts and ultimately guide programmatic decision making.

This article analyzes the relationships formed during this single training workshop for sampling wild bats and conducting laboratory analysis. We also discuss a prior analysis of scientific collaborations formed during a disease outbreak and idea of transformative science (1). This prior analysis found that research collaborations with the potential to be transformative depend on human interactions and cooperative foundations within disciplines, effective collaborative mutualism across disciplines, and a communication process that enables knowledge synthesis across diverse perspectives. We demonstrate the evolution of these processes through analysis of workshop participants using a system dynamics model. We show that the outcomes from the workshop can not only be measured but also weighted for relative importance to the team and workshop leaders using a ROR metric. This approach will enhance the efficacy of future collaborative efforts by weighting variables that are not usually considered for success.

## Materials and Methods

### Approach

Similar to bibliometric analysis of scientific co-authorship networks (9), we analyze scientific networks represented through proposals, meetings, posters, and other metrics of scientific output. In contrast to co-author network analysis that represents a series of complex-evolving networks after a period of time has elapsed, our analysis tracks and measures network dynamics during the earliest phase of collaboration. We visualize and model the dynamic and the structural mechanisms that govern the evolution of the complex system of scientific collaborations initiated during a bat-borne pathogen biosurveillance training workshop held in 2014. As an example of the long-term outcomes from an initial group of researchers, we discuss the outcomes from the relationships that developed from the Hantavirus outbreak in New Mexico in 1993. These two events represent the end members of a spectrum of collaborations between scientists. The first event involved deliberate and intentional selection and mixing of potential collaborators, fostering cross-fertilization of ideas across disciplines; the second generated new collaborations due to crisis and circumstance.

### The Workshop

In June 2014, members of the Laboratory of Virus Evolution in the Program of Emerging Infectious Diseases at Duke-NUS Medical School in Singapore designed and taught a 2-week course on infectious diseases in bats (Bat Borne Pathogen Surveillance Workshop). Participants were from the Philippines, Taiwan, the Republic of Georgia, Thailand, Jordan, and the USA and were country partners with the U.S.-based Cooperative Biological Engagement Program (CBEP), a component of the Defense Threat Reduction Agency’s Cooperative Threat Reduction program. The training, which was initiated and funded by CBEP, leveraged the expertise from the Program in Emerging Infectious Disease at Duke-NUS Medical School, to provide a comprehensive course that covered a wide variety of topics on EIDs in bats. While the primary goal for the bat-borne pathogen training was to increase the capacity and capabilities for biosurveillance in partner countries, a secondary and ultimately perhaps more important goal was to develop a sustainable network for researchers interested in One Health. The participants were able to build on the relationships forged at during the training support and leverage these into research collaborations.

Participants were primarily virologists and laboratory-based scientists, and we provided a course that captured the basics of bat biology, ecology, field surveillance, laboratory screening, and phylogenetic analysis. Thirteen individual lectures were given over the course of 2 weeks. Also included were four field trips to trap and process bats in the field and gain an understanding of the work flow from field to laboratory to analysis (*field to phylogenies*). The final day of the training focused on molecular evolution, sequence alignment, selecting evolutionary models, and phylogenetic tree reconstruction with both lectures and laboratory practicals. All participants were vaccinated for potential zoonotic pathogens, such as rabies, and all animal work was approved by the NUS IACUC (B01/12). A day-long, interactive workshop demonstrated the necessity of a cohesive approach to zoonotic pathogens.

An interactive session was held near the end of the 2-week training and focused on two case studies, the historical Sin Nombre Hantavirus outbreak, which occurred in the Four Corners area of the United States, and the present day Middle East Respiratory Syndrome Coronavirus (MERS CoV) outbreak, which originated in Saudi Arabia but is currently presenting cases worldwide. The goal of the session was to utilize the historic Hantavirus outbreak case study to illustrate how a collaborative approach to problem solving can lead to transformative science. Building upon this example, participants were guided through an exercise where they applied that concept to developing mechanisms (and relationships) to answer questions about the transmission of MERS CoV that remain unanswered today. Finally, the relationships and potential collaborations identified that day and forged during an intensive 2-week training course in which they lived, worked, and learned together were then tracked and monitored over the next year as the participants returned to their respective countries and institutes.

The eight researchers who came together for this training and workshop represented eight countries in three regions. Like the original, multidisciplinary group of scientists who solved the mystery of Hantavirus transmission in 1993 (1), they brought divergent ideas, scientific cultures, and skillsets together for the purpose of solving one very challenging global health problem. By asking scientific questions across those diverse scientific disciplines and cultures, the potential for unexpected answers can arise. And those answers, as well as the process that generated them, have the potential to drive the creation of new paradigms for scientific problem solving. The transformative science recognized and documented in the discovery of Hantavirus in 1993 (1), which solved a single problem causing a previously unrecognized transmission mechanism, can now be fostered and deliberately directed to aid in solving complex health security issues. But in order to validate this process and demonstrate its effectiveness to scientists, program managers, and policy makers, an analytical metric first had to be developed.

### System Dynamics of Scientific Collaborations

We developed an aggregate level system dynamics model, which considered the scientific training event (Bat Borne Pathogen Surveillance Workshop) as a resource-limited, finite capacity system operating under control constraints. The scientific social network or “system” that started with the Bat Borne Pathogen Surveillance Workshop were modeled as ordinary differential equations and evaluated in the system dynamics software tool, Vensim™. Mathematically, the basic structure of a system dynamics computer simulation model in Vensim™ is a system of coupled, non-linear, first-order differential (or integral) equations. The Bat Borne Pathogen Surveillance Workshop was modeled as a single starting event that evolved outwards as additional relationships unfolded. The system dynamics model, in this case, is only used to weight the different outcomes that came from a particular relationship and keep track of the endpoints with an overall index. The system dynamics model developed would be considered one of the simplest ways to weight, calculate, and visualize a scientific social network stemming from an event.

Relationships were identified by connections made at the initial training event that led, sometimes by multiple steps or subsequent connections to specific deliverables or outcomes, such as collaboration on a proposal, a paper, or a training event (Table 1). Weights were given for deliverables based on a scale of 1–5 for potential low to high value. Scientists or organizations were considered connected if they authored a paper, proposal, or other deliverable together. It is important to note that the weights assigned are based in part on programmatic value and effectiveness in achieving the CBEP goal of providing capabilities to sustain biosurveillance, biosafety, and safe research objectives to strengthen defenses against the threat of infectious disease. Other programs with differing goals and objectives might assign weights differently and even identify other deliverables to track and measure while employing this system of analysis.

**Table 1:** **System dynamics model weighting for Bat Borne Pathogen Surveillance Workshop I**.

Stakeholders	Weighting	Result *N* (weighting)
Countries	2 for country partner	14(2) = 28
	1 for non-partner	1
Collaborators, organizations, and institutes	Weighted the same (1)	17
**Deliverables (D_i_)**		
Proposals	1–5 (low to high potential value)	7(5) = 35
Project development grants (PDG)	1–3	2(3) = 6
Publications/posters	1–5	3(3) + 1(5) = 14
Curriculum	1–5	1(4) = 4
Training events/conferences	1–5	5(5) = 25
Capability (assay, standard operating procedures)	1–5	0
Organized meeting with stakeholder and country partners	1–5	10(3) = 30
**Event metric (*S* + *D*):** (*S*) = stakeholders = countries + collaborators; (*D*) = deliverables = Σ*D_i_*		Total = 114

*Weights are on a scale of 1–3 or 1–5 for relative importance or status of deliverable*.

### Measuring the Return on Relationships

The eight researchers from the Philippines, Taiwan, the Republic of Georgia, Thailand, United States, and Jordan who participated in the workshop each brought unique relationships with institutes, universities, and government offices that could be leveraged for collaborations as deemed appropriate and useful. Each participant had different background and experience in the laboratory and field experience working with animals. We identified a total of 23 measurable deliverables that were the outcomes of the workshop. Nine separate proposals were developed between the participants in the workshop, with smaller project development grants (PDG) being weighted lower than full 3-year proposals for research. Four publications or poster presentations at conferences were developed from the workshop and longer, more developed publications or larger scientific conferences were weighed higher. Even though the original cohort was from seven countries, including Singapore, 12 countries were represented in the outcomes, illustrating the immediate and rapid expansion of the initial collaborative network. One educational curriculum was developed from the workshop, which covered the lessons learned from the Hantavirus outbreak and discovery in 1993 (Table 1). Over 20 institutes, universities, or government organizations were brought together through the outcomes from this workshop, and that list continues to grow. Three working groups or research-coordinated networks (RCN) modeled after the National Science Foundation’s RCNs in the United States have been formed. The original training curriculum from the Singapore event has been adapted and expanded for use in two other regions where the participants have begun to tackle the problems and questions presented by the MERS CoV outbreak. Two additional Bat Borne Pathogen Surveillance Workshops with the same cohort have been planned with additional countries being trained in Bat and Camel Pathogen Surveillance, with an emphasis on MERS CoV. For the initial Bat Pathogen Surveillance Workshop, the final value for an index of the number of weighted outcomes and potential relationships that resulted from the workshop was 115 (Table 1).

## Discussion

### The Value of a Single Workshop

A commonly used, numbers-based metric for trainings is the number of people trained or “butts in seats.” However, how do we fit the measurement of collaborative efforts in multidisciplinary research and training efforts into a framework of results-based metrics such that the value of ROR can be quantified? How do we demonstrate that investment in the creation and fostering of relationships that are long-lasting and committed to solving problems will result in transformational outcomes that ultimately contribute to reduction of the threat of infectious disease?

Social and collaboration networks are often invisible. That is, they are so interwoven into the fabric of our lives, that they are not observable. As it is often stated, what is not measureable does not exist. As a result, the importance of scientific social networks is often underappreciated and unknown, and consequently undervalued. As with any metric, once scientific social (relationship-based) networks can be identified and measured, emphasis on objectives to increase the metrics can increase. For example, motivation to improve measured results can lead to an increased emphasis in the quality of resulting and differentially weighted deliverables.

For the initial Bat Borne Pathogen Surveillance Workshop, the value of the index or metric was 115, which in the same way as other indices, is not useful without comparison to other events calculated in the same manner. The usefulness of this social network metric lies in the comparison to relevant events to help discern the relative value of the time and resources to organizations. In market-driven business engagements, there are primarily two sides to a value proposition, namely value for the supplier and value for the customer (10). In cooperative engagements, with the shared mission of reducing the threat of diseases, the value in sharing information is realized by improvements in the capability to better detect, diagnose, and report on diseases. With each deliverable, such as a curriculum, manuscript, or training event, communication and trust between the cooperative parties is increased and the value to the shared mission is increased. Within an organization, the benefit for estimating the ROR for an event is in comparing to other events to discern the relative value of often declining, resources. Another benefit for measuring the outcomes from an event is that is can be used in communicating the return of investments to all of the stakeholders in a cooperative engagement. This article demonstrates how a cooperative relationship can be seen as a mutual investment, where reciprocal return on this investment in the relationship can be assessed, and moreover, how this investment pays off as ROR for all stakeholders in the shared mission of reducing the threats of infectious diseases.

### Scientific Collaboration and Transformative Science

The One Health initiative explicitly states that interdisciplinary and cross-sectional approaches are required for the prevention, surveillance, monitoring, control, and mitigation of EIDs. The terms multi- and interdisciplinary describe research efforts that incorporate a variety of disciplinary perspectives with varying degrees of integration across perspectives (11). Collaboration between two researchers in different disciplines toward a more comprehensive understanding of a particular infectious disease is an example of multi- or interdisciplinary collaboration. In contrast, transdisciplinary collaboration extends beyond changing our understanding to invoke outcomes beyond the research arena, such as changing the way a disease is diagnosed or treated. Transdisciplinary collaboration explicitly seeks to have transformative outcomes for individuals, professions, or society as a whole, and accomplishes this by intentionally incorporating people and activities designed to convey research results beyond the research community. Hence, the One Health initiative call for interdisciplinary and cross-sectional approaches is an explicit call for transdisciplinary research that has transformative outcomes in all aspects of disease control. And most importantly, the need to support activities designed to foster transdisciplinary collaboration is clear if the collective effort to solve broad global health problems is to succeed.

### The Hantavirus Outbreak: An Example

In 1993, a series of unexplained deaths in the American Southwest prompted an immediate response from scientists representing a variety of disciplines and institutions, who ultimately discovered critical linkages between the El Niño Southern Oscillation (ENSO), increased precipitation, vegetation primary productivity, deer mouse (*Peromyscus maniculatus*) populations, and Sin Nombre Virus (12). This transdisciplinary group produced a wealth of outstanding science with immediate, high societal impact.

In a previous analysis of a scientific collaboration that resulted from the Hantavirus outbreak in New Mexico in 1993, it was shown that transformative scientific collaboration depends on human and material foundations within disciplines, effective collaborative mutualism across disciplines, and a learning process that enables knowledge synthesis across diverse perspectives (1). This outbreak prompted new collaborations between clinicians, public health professionals, epidemiologists, pathologists, molecular biologists, and mammalogists and transformed the research of the scientists who were involved, creating new paradigms in the zoonotic infectious disease community and leaving a lasting, positive impact on medical community practices. Specifically, Pennington et al. (1) found that being thrust into disorienting activities associated with rapidly changing information from a variety of disciplinary perspectives initiates transformational learning and postulated that this is a key mechanism for generating new, creative scientific understanding. They argued that deliberate involvement in transdisciplinary activities in the absence of crisis could potentially result in similar outcomes. This would occur through exposure to unfamiliar concepts, methods, and perspectives that invoke transformational learning and generating new, innovative ideas. Indeed, Pennington (13) found that researchers involved in interdisciplinary workshops were motivated to participate because the exposure to other disciplinary perspectives was having a high impact on their own research and was leading to the generation of creative research ideas (13). Transformational science begins with transforming the perspective of an individual scientist, and this is in direct response to exposure to perspectives besides one’s own. A single workshop can have enormous impact if even one potentially transformative idea is generated. This impact is not measurable by any kind of co-author analysis.

In the Hantavirus example, new collaborations formed as a result of the outbreak and a transdisciplinary team was quickly assembled. This was accomplished because the outbreak was local, relevant expertise could be found locally and in the nearby region, and others were able to rapidly converge on the area from elsewhere in the United States. As observed in the recent Ebola outbreak in 2014, global connectivity makes it increasingly likely that infectious outbreaks are not confined to a single locality and easy access to relevant expertise is not assured. A core ideal of CBEPs is one of transparency and collaboration that builds trust between partner countries to facilitate data and information sharing to allow each partner to better respond to infectious disease threats. Transparency and trust are key attributes of successful collaborations that are not easily generated between individuals or organizations (14, 15). Distance effects are very real despite global communications (16). Studies of science teams identified the importance of face-to-face interactions early in a collaborative group (17, 18). These early interactions build transparency and trust and can greatly improve later interactions that are not face-to-face. The Internet has the potential to enhance collaboration among researchers by supporting technical applications that coordinate numerous and complex real-time interactions and can facilitate the rapid dispersal of information between researchers (19) – if transparency and trust are in place (20).

In a study of 699 people working in groups from two to five in a wide variety of settings, Woolley et al. (21) found that the best predictors of creative outcomes were the degree of turn-taking in team interactions and team members’ ability to read facial expressions. Intelligence of the team as a whole or of any individual on the team was less important. They proposed a new measure of collective intelligence as a predictor of the creativity of group outcomes (21). A single workshop can begin to generate collective intelligence between participants that is difficult to obtain without the turn-taking and interpretation of facial expressions and body language that occurs in person.

### Lessons Learned

The two events differ in that the outbreak response to Hantavirus occurred prior to wide use of the internet and hence, the sustained collaboration that resulted was enhanced by geographic proximity of collaborators. With the ubiquity of the internet modern collaborations are not limited by geography. The Internet has the potential to enhance collaboration among researchers located across the globe by supporting technical applications that coordinate numerous and complex real-time interactions and can facilitate the rapid dispersal of information between researchers (19). Indeed, a core ideal of CBEPs is one of transparency and collaboration that builds trust between partner countries to facilitate data and information sharing to allow each partner to better respond to infectious disease threats.

Using the two examples of the Bat Borne Pathogen Surveillance Workshop and the Hantavirus outbreak response and the resulting networks, what were the lessons learned for increasing the return of relationships? What are things we can do to increase the ROR metric? And are there things that can hurt relationships and break down bridges? From our experience and talking to participants in both events, the following things helped foster stronger relationships between the scientists.

- Having a discussion between all participants on the value of collaborative relationships and the relevance to reducing the threat of infectious diseases helped participants keep a conscious effort for maintaining the collaboration.
- Consistently highlighting and writing down collaborative ideas between participants as they occur resulted in leaving with the event with a collection and outline of collaborative ideas.
- As a last exercise in the event, having participants review the collaborative ideas and develop potential next actions as well as deliverables (metrics) along the way for each party.
- Providing an infrastructure either in funding for additional meetings or an online social site for the group to help strengthen and forge the sustainability of the relationships.
- Finding and giving examples of funding opportunities or supporting scientific collaborations and highlighting deadlines for application.
- Allowing for social activities as part of the event helps foster connections and helping maintain potential future opportunities to meet or connect. Providing opportunities to maintain the relationships over time only builds on the initial connection.
- Not doing any of the above suggestions can impede the success of a research collaborative network, but also forcing participation can backfire. Presenting the data on the positive application of professional networks and continuing to provide opportunities for strengthening the network can invigorate the group to be enthusiastic for working together in the future.

Here, we show that visualizing and quantifying scientific social networks that develop from a specific event can be useful in estimating the impact of collaborations on a field or mission, such as reducing the threat of infectious diseases. A primary goal for cooperative engagement programs is to advance a field together in research or education by supporting groups of investigators to communicate and coordinate their research and training and educational activities across disciplinary, organizational, geographic, and international boundaries. Established relationships may be paramount in preventing the next pandemic.

## Author Contributions

All authors listed, have made substantial, direct and intellectual contribution to the work, and approved it for publication.

## Conflict of Interest Statement

The authors declare that the research was conducted in the absence of any commercial or financial relationships that could be construed as a potential conflict of interest.
